# Constructing identity and social capital on Facebook: a feminist digital sociology of Bangladeshi women

**DOI:** 10.3389/fpsyg.2025.1634395

**Published:** 2025-08-11

**Authors:** Md Nurus Safa, Tahera Akter, Nusrat Jahan

**Affiliations:** School of Media and Communication, Shanghai Jiao Tong University, Shanghai, China

**Keywords:** Facebook use, social capital, identity construction, Bangladeshi women, digital feminism, network diversity, PLS-SEM

## Abstract

Facebook has become a crucial digital platform for women in Bangladesh, facilitating novel avenues for interaction, support, and identity formation within a patriarchal societal framework. This mixed-methods study examines the role of Facebook activity in the construction of social capital and the development of identity among Bangladeshi women. The study utilizes survey data from 357 women, analyzed by Partial Least Squares Structural Equation Modeling (PLS-SEM), to investigate the relationships between Facebook usage, network size and diversity, bonding and bridging social capital, and self-identity outcomes. The findings indicate that increased Facebook participation is a strong predictor of both network variety (*β* = 0.72) and size (*β* = 0.55), which subsequently improve bonding (R^2^ = 0.61) and bridging (R^2^ = 0.43) social capital. These network-derived social resources enhance the dynamism of identity creation (R^2^ = 0.425), particularly among women with varied digital connections. Qualitative insights derived from 15 comprehensive interviews elucidate these findings: Participants characterize Facebook as a dual-faceted platform broadening perspectives and facilitating self-expression, while also limited by monitoring, conservative standards, and calculated self-presentation. The research incorporates Social Capital Theory, Social Identity Theory, and Goffman’s dramaturgical framework to illustrate how digital environments simultaneously replicate and challenge gendered power dynamics. These findings enhance feminist digital sociology and ICT4D literature by elucidating how social media platforms facilitate empowerment, connectivity, and identity for women in the Global South.

## 1 Introduction

Social media platforms have become essential to daily life globally, facilitating new opportunities for interaction, community development, and self-expression. Facebook continues to be the preeminent social networking platform in South Asia, particularly in Bangladesh, where millions of women have registered in recent years (Chowdhury J. N. and bin Ahsan, 2024; Chowdhury N. and bin Ahsan, 2024). In conservative nations, online platforms frequently provide women with essential avenues for engagement beyond the confines of historically limited offline environments (Fatima et al., 2025). Nonetheless, the ramifications of Facebook usage on women’s social capital and identity are still insufficiently explored in Global South contexts. Social capital encompasses the resources and advantages obtained from social connections. The classical view differentiates between bonding social capital intimate, robust connections usually comprising family and trusted friends and bridging social capital, characterized by weaker links that connect individuals across diverse social divisions (Lin, 2001; Putnam, 2000). Theories posited by Bourdieu, Coleman, and Putnam elucidate the capacity of social networks to provide emotional support, information, and access to opportunities (Page-Tan, 2021).

Identity formation in the digital era can be analyzed from social psychology and sociological perspectives. Social Identity Theory (Tajfel and Turner, 1979) elucidates how group connections influence individuals’ self-concept via categorization, identification, and comparison processes. Goffman’s dramaturgical approach (1959) elucidates how individuals enact social roles, presenting curated impressions on a metaphorical “front stage” while hiding aspects on the “backstage.” These ideas are particularly relevant in the Bangladeshi setting, where women must maneuver through intricate cultural norms and audience expectations while participating online (Banet-Weiser, 2018; Wang et al., 2020).

Recent research indicates that social media can both empower and limit women in traditional countries. Platforms such as Facebook empower women to contest gender norms by providing avenues for self-expression, knowledge dissemination, and community engagement (Choi, 2024; Salam, 2020). Women can establish bridging links through online organizations, increasing exposure to novel ideas and collaborative action. Conversely, apprehensions regarding surveillance, harassment, and societal criticism frequently reduce their online engagement (Chan, 2018; Hakimi et al., 2024). Research in Bangladesh indicates that the apprehension of online harassment compels numerous women to self-censor or adopt meticulously controlled self-presentation methods (Banet-Weiser, 2018; Klose and Jebin, 2024). One respondent stated that Facebook enables women to “keep their mouths shut” while attempting to project an ideal image in public, underscoring the ongoing patriarchal scrutiny in digital environments (Wang et al., 2020). Consequently, Facebook serves as a double-edged sword for Bangladeshi women: it expands their social and informational vistas while simultaneously mirroring offline power imbalances and cultural limitations.

### 1.1 Research gap and objectives

A significant portion of the literature concerning social media, social capital, and identity originates from Western contexts or employs gender-neutral viewpoints (Couldry and Mejias, 2019; Raza et al., 2016). There is an increasing necessity to prioritize women’s lived experiences in the Global South, where gender norms, digital disparities, and cultural expectations significantly influence platform utilization (Tasnima et al., 2024). Recent scholarship in feminist digital media highlights the necessity for intersectional and context-specific research regarding women’s engagement with digital platforms in non-Western societies, particularly under conditions of surveillance, patriarchy, and platform capitalism (Banet-Weiser, 2018; Fatima et al., 2025; Mei and Symaco, 2022). While there is an expanding body of literature on online identity and social media in the Global North, there remains a paucity of empirical research examining how women in the Global South, particularly within Muslim-majority patriarchal contexts such as Bangladesh, utilize digital platforms for self-expression and the formation of social capital. This gap highlights the necessity for feminist research that contextualizes digital identity within the daily experiences of structural inequality, gendered digital divides, and sociocultural limitations.

This study examines the involvement of Bangladeshi women using Facebook. This research, grounded in Social Capital Theory, Social Identity Theory, and Goffman’s self-presentation model, examines the impact of Facebook usage on the size and diversity of women’s online networks, the translation of these characteristics into bonding and bridging social capital, and the collective contribution of these factors to identity construction in digital environments. A mixed-methods approach is employed, integrating a quantitative survey evaluated via structural equation modeling (SEM) with qualitative in-depth interviews to obtain comprehensive insight. The primary study question is: (1) To what extent does Facebook usage augment the bonding and bridging social capital of Bangladeshi women? (2) How is network size associated with Facebook involvement and social capital outcomes? (3) How can social capital generated by Facebook assist to the formation of women’s identity and self-presentation? (4) What obstacles or contextual elements influence these processes?

This study critically analyzes Facebook’s influence on women’s social relationships and self-perceptions within a patriarchal Bangladeshi society. This research highlights the lived experiences of women in a developing, Muslim-majority nation, so enhancing the worldwide comprehension of the social ramifications of digital media. It provides insights pertinent to feminist digital sociology and ICT4D, demonstrating how digital platforms may both perpetuate and redistribute power in gendered manners across cultural and technological boundaries. This study enhances the area of feminist digital sociology by providing empirical evidence from a Global South context, where cultural, religious, and gendered restrictions influence women’s digital agency. This research enhances understanding by integrating Social Capital Theory, Social Identity Theory, and Goffman’s dramaturgical model to reveal how Bangladeshi women navigate identity and community on Facebook. It provides a contextual, gender-aware examination that broadens the prevailing Western-centric narrative on social media and identity.

## 2 Literature review and theoretical framework

### 2.1 Social media and social capital

Social capital denotes the advantages individuals derive from their social networks, encompassing support, information, trust, and cooperation (Lin, 2001; Putnam, 2000). Researchers differentiate between bonding social capital, characterized by robust connections within homogeneous, close-knit groups, and bridging social capital, which entails weaker links that link individuals across varied social divisions (Ellison et al., 2007; Page-Tan, 2021). Bonding capital furnishes emotional support and solidarity, whereas bridging capital facilitates access to new information and opportunities, including job or education (Phua et al., 2017). Furthermore, social capital cultivated by social media has been associated with psychological well-being and thriving, especially when facilitated by significant online interactions (Gudka et al., 2021).

Putnam (2000) elucidated the distinction between bonding and bridging in his examination of communal life, asserting that both are essential for a robust society: bonding fosters social glue, whilst bridging functions as social WD-40, promoting innovation and connectivity. In Bangladesh’s patriarchal society, familial and intimate friendships are typically essential for women due to insufficient institutional support (Fatima et al., 2025). Bridging relationships can be transformative by providing women with new knowledge, various viewpoints, and expanded social resources, which is especially crucial in contexts where gender norms limit women’s mobility and public engagement (Salam, 2020; Tasnima et al., 2024).

Social networking services such as Facebook have transformed the dynamics of social capital development. Facebook allows users to sustain extensive and diverse networks encompassing both strong and weak connections. Preliminary studies, including Ellison et al. (2007), indicated that regular Facebook usage among U.S. college students correlated with enhanced bridging social capital. The Facebook Intensity Scale, created by Ellison et al. (2007), demonstrated that profound activity on the network enhanced informal social support and access to information.

Subsequent research indicates that Facebook can concurrently strengthen bonding links (by regular interaction with close connections) and broaden bridging ties across geographic and social divides (Raza et al., 2016; Smock et al., 2011). However, it’s not enough to have a lot of friends to have real social capital. As Granovetter (1973) pointed out in his theory of weak ties, having connections with people from different fields, ideologies, and demographics is important for getting non-redundant information and building bridges. Moreover, the quality of online contacts is significant: weak ties can only provide bridging advantages if they are defined by trust and reciprocity. Previous studies indicate that a high quantity of Facebook friends does not inherently result in significant social capital. Network diversity and relational quality are essential factors influencing bridging effectiveness (Ellison et al., 2011; Granovetter, 1973; Haythornthwaite, 2002). This context is essential for analyzing the experiences and perceptions of Bangladeshi women regarding digital connections. Although bonding and bridging social capital offer unique advantages, the significance of network variety and the reliability of weak relationships is essential for facilitating substantial social growth (Granovetter, 1973; Williams, 2006). Granovetter’s (1973) concept of weak ties, which refers to connections outside of close-knit groups, posits that they are essential for obtaining non-redundant information, discovering innovative chances, and gaining broader perspectives. On Facebook, these relationships frequently commence as latent connections between persons who are linked yet exhibit low interaction (Haythornthwaite, 2002). Latent ties can transform into mobilizable ties by frequent exposure, shared material, or collective group involvement, enabling activation for advice, support, or collaboration (Ellison et al., 2011; Phua et al., 2017).

In Bangladeshi women, this evolution often transpires through particular platform habits, including commenting on others’ posts, participating in group discussions, or disseminating culturally significant content (Tasnima et al., 2024). In Bangladeshi women, this evolution frequently occurs through specific platform behaviors, such as commenting on posts, engaging in group discussions, or sharing culturally relevant content. Women often begin by observing information in women-only Facebook groups, later progressing to discussions that build trust and enable a broader range of topics. Micro-interactions, especially within semi-anonymous or shared-interest groups, provide a low-risk means of bridging capital, thereby extending social resources beyond familial or neighborhood-based bonding. Patterns have been identified in similar contexts, indicating that women employ digital platforms as environments for gradual trust-building (Castillo de Mesa et al., 2020; Yin and Zhang, 2024). These shifts depend on the perceived safety, communal standards, and reciprocity within digital environments.

### 2.2 Social capital theory in a gendered context within global south

Conventional social capital theory (Lin, 2001; Putnam, 2000) predominantly neglects women and digital environments. Recent feminist and development studies have investigated how women in patriarchal settings utilize social media to cultivate social capital (Fatima et al., 2025). In Bangladesh, institutional obstacles including restricted schooling, mobility limitations, and societal monitoring hinder women’s capacity to establish offline networks (Chowdhury N. J. and bin Ahsan, 2024; Chowdhury N. and bin Ahsan, 2024; Tasnima et al., 2024). Facebook and mobile internet have commenced transforming this landscape by facilitating women’s connections beyond conventional environments.

Studies demonstrate that Bangladeshi women using Facebook to establish peer communities and obtain informational and emotional support (Fatima et al., 2025). Women-exclusive Facebook groups function as secure environments for sharing advice and mobilizing resources, thereby augmenting bridging capital (Castillo de Mesa et al., 2020), as referenced in Tasnima et al. (2024). Nonetheless, these digital connections are entrenched in systemic inequalities. Sultana (2010) observed that over dependence on familial bonding capital can alienate women from wider society resources. Homophily, the inclination to connect with similar individuals, may cause Facebook users to reinforce existing social boundaries instead of overcoming them (Labben, 2022).

Furthermore, although disparities in digital access remain especially among women in rural regions those who are digitally engaged exhibit considerable potential for developing social capital via Facebook. This study examines the experiences of women who are active Facebook users, investigating how their engagement on the platform influences their social networks and identity, amidst the broader challenge of digital inequality (Hakimi et al., 2024). The study anticipates a favorable correlation between the Facebook usage of Bangladeshi women and their network characteristics in everyday life, based on previous findings (Ellison et al., 2007; Phua et al., 2017).

*H1*. Facebook involvement is positively associated with network diversity.

*H2*. Facebook involvement is positively associated with network size.

*H3*. Network diversity is positively associated with bonding social capital.

*H4*. Network diversity is positively associated with bridging social capital.

*H5*. Network size is positively associated with bonding social capital.

*H6*. Network size is positively associated with bridging social capital.

### 2.3 Theoretical framework of social identity

Social Identity Theory (SIT) elucidates how individuals characterize themselves through group affiliations and pursue a favorable self-concept through social comparison (Tajfel and Turner, 1979). It delineates three fundamental processes: classification (establishing group borders), identification (embracing group norms), and comparison (assessing one’s group in respect to others).

Bangladeshi women engage in many networks on Facebook that shape identity formation. Participation in parenting forums promotes conventional roles (motherhood), but involvement in entrepreneurial or activist groups facilitates the development of alternative identities (Banet-Weiser, 2018; Wang et al., 2020). The conflict between these responsibilities is heightened in conservative nations where online exposure attracts attention.

Women frequently navigate various identities, showcasing culturally accepted personas in public forums while participating in more subversive discussions within private groups (Smidi and Shahin, 2017). This balancing effort illustrates how digital environments facilitate empowerment while concurrently subjecting users to social sanctions, contingent upon group norms and approval (Chan, 2018). Consequently, SIT offers the psychological and cultural foundations essential for comprehending how Facebook influence’s identity development and network formation among Bangladeshi women. Therefore, this study proposes the following hypotheses:

*H7*. Network size is positively associated with identity construction.

*H8*. Network diversity is positively associated with identity construction.

We differentiate between identity construction and identity performance to maintain conceptual clarity. Identity construction involves the gradual formation of a cohesive self-concept, shaped by social interactions and individual reflection (Luyckx et al., 2008). In contrast, identity performance entails the strategic presentation of the self to an audience, influenced by social norms, expectations, and platform affordances (Goffman, 1959). Within Facebook, users often engage in processes that intersect as they curate their profiles and interactions to explore their identities and manage perceptions across various social groups.

### 2.4 Theory of self-presentation by Goffman

Goffman (1959) defined identity as a performance enacted in public (“front stage”) and private (“back stage”) contexts. Social media platforms such as Facebook serve as a principal arena for impression management. Women in Bangladesh frequently construct profiles that conform to societal norms, disseminating information that embodies familial values, educational attainment, and religious practices (Klose and Jebin, 2024). Simultaneously, they utilize secret groups or pseudonymous identities to articulate dissenting opinions or solicit assistance on sensitive subjects such as mental health, gender-based violence (Bharathi, 2023; Hou, 2020).

Goffman’s theory elucidates the impact of self-presentation methods on social capital and identity. The public exhibition of conformity may aid in maintaining bonding capital, whereas private conduct within restricted groups promotes identity discovery and bridging capital. However, this compartmentalization may lead to superficial relationships and internal conflict, as individuals navigate competing roles and identities (Couldry and Mejias, 2019). In Bangladesh’s patriarchal framework, digital self-presentation is influenced by the convergence of cultural norms and the technological capabilities of platforms such as Facebook. On Facebook, women meticulously construct their public personas to embody culturally acceptable indicators, including familial values, religious commitment, or scholarly accomplishments, in order to preserve social legitimacy and evade reputational repercussions within scrutinized networks (Klose and Jebin, 2024; Sultana, 2010). These performances are frequently shaped by familial trust dynamics and the significant societal repercussions of public deviance in gender-conservative settings. Simultaneously, Facebook’s technical attributes, like restricted groups, privacy filters, and selective audience settings, facilitate a mode of backstage navigation, allowing women to investigate less conspicuous and occasionally subversive avenues of self-expression (Chan, 2018; Smidi and Shahin, 2017). Participation in anonymous women-only groups enables individuals to seek emotional support or express concerns regarding matters such as mental health or domestic violence. Consequently, online identity management necessitates a delicate equilibrium: preserving bonding capital via socially acceptable profiles while concurrently fostering bridging capital via secure, diversified networks. This dual navigation highlights the interplay between digital affordances and established gender norms, demonstrating the coexistence of agency and restriction in the Facebook usage of Bangladeshi women (Couldry and Mejias, 2019; Tasnima et al., 2024).

### 2.5 Facebook and women’s digital identity in non-Western contexts

The majority of research regarding Facebook and identity predominantly examines Western contexts, frequently neglecting the gendered intricacies of digital identity in the Global South (Fatima et al., 2025; Wajcman, 2010). In Bangladesh, Facebook functions as a semi-public arena where women navigate a precarious equilibrium between empowerment and vulnerability (Chowdhury N. J. and bin Ahsan, 2024; Chowdhury N. and bin Ahsan, 2024; Tasnima et al., 2024). For some individuals, Facebook serves as the sole venue for identity inquiry beyond the confines of the domestic realm. Research indicates that Facebook provides women with avenues for community-building, education, and political participation, but frequently moderated by prudence and self-censorship (Hakimi et al., 2024; Mamun, 2024). Harassment, digital exclusion, and monitoring continue to exist, influenced by wider socio-economic and technological inequalities (Rahman, 2023).

This research enhances feminist literature by offering empirical data from Bangladesh, a context where patriarchy, digital inequity, and cultural restrictions interact in distinctive ways. We conceptualize Facebook not merely as a communication platform but as a contentious space for identity construction, negotiation, and resistance. This corresponds with extensive research highlighting that digital identity is a socially negotiated and dynamic construct within data-driven societies (Rowland and Estevens, 2024). The framework of Facebook particularly its functionalities such as customized privacy settings, closed groups, and symbolic elements like “likes” and “reactions” significantly influences how women in Bangladesh navigate their digital identities. These platform-specific capabilities enable users to adjust their visibility and strategically influence impression formation in ways that conform to or contest societal norms. The ability to post in private or women-only communities offers a semi-anonymous environment where women can articulate personal opinions, get assistance, or disseminate content without the risk of public scrutiny (Chan, 2018; Chowdhury N. J. and bin Ahsan, 2024; Chowdhury N. and bin Ahsan, 2024). Likewise, regulating the audience for posts (e.g., “friends only,” “custom,” or “only me”) allows women to showcase certain facets of their identity to distinct audiences reinforcing Goffman’s (1959) concept of front-stage and back-stage performances. Even minimal interactions, such as receiving “likes” or encouraging remarks, might provide emotional validation, hence strengthening identity assertions in digital environments (Banet-Weiser, 2018; Papacharissi, 2015). Consequently, Facebook’s architecture is not impartial; it promotes selective self-expression and identity exploration while concurrently imposing limitations through social visibility, algorithmic filtering, and normative norms.

### 2.6 Integrative framework

**Figure 1:**
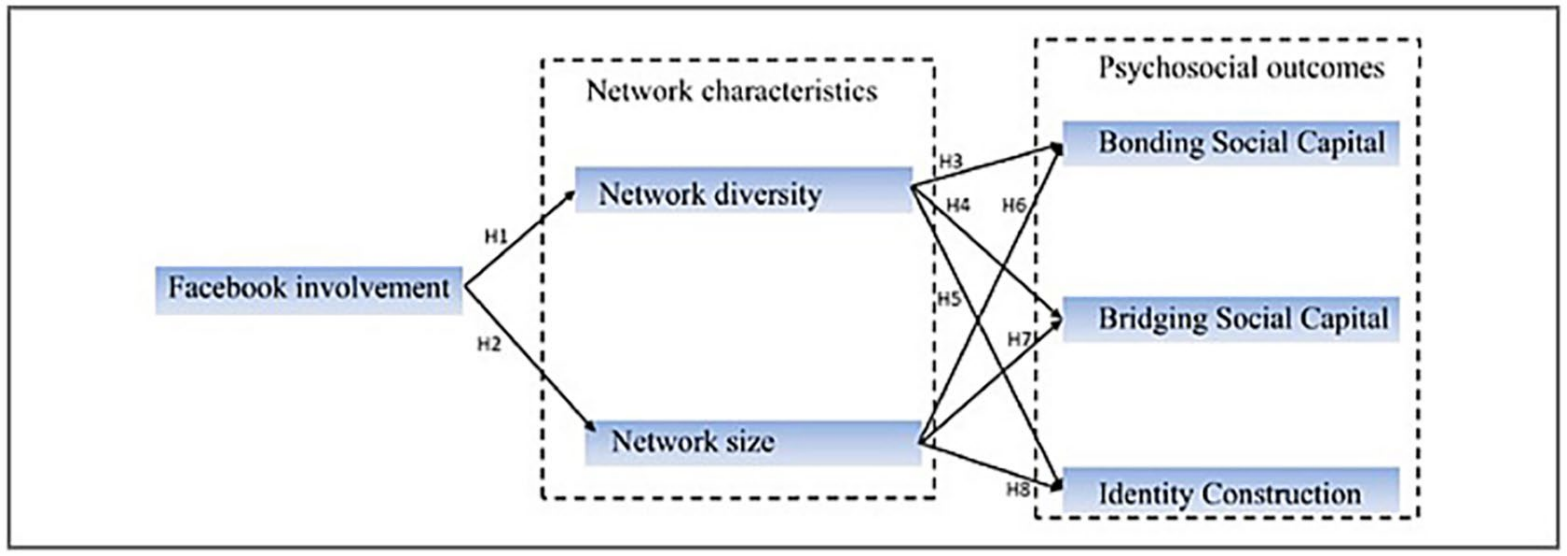
Conceptual framework and research hypotheses model.

This research synthesizes Social Capital Theory, Social Identity Theory, and Goffman’s dramaturgical framework to examine the influence of Facebook activity on network structure, social capital, and identity formation. We propose that Facebook usage expands network size and diversity, thus augmenting bonding and bridging capital. Social capital is posited to significantly influence the relationship between network characteristics and identity outcomes. Increased network variety is expected to facilitate more dynamic identity formation through interaction with diverse individuals and novel concepts, whilst robust bonding capital provides emotional support for identity validation (Benitez et al., 2020). Nonetheless, these interactions are also shaped by contextual limitations, including gender conventions, computer literacy, and platform capabilities. The impact of homophily may constrain the bridging capacity of diverse networks. Facebook facilitates broader connections; however, users frequently prefer interactions with individuals who share similar values or identities, thereby reinforcing established social boundaries (Labben, 2022). This dynamic should be recognized as a limitation in the interpretation of network diversity’s role in the expansion of social capital. The conceptual Framework comprises eight hypotheses. H1–H2 analyze the impact of Facebook engagement on network attributes, specifically diversity and size. H3–H6 examine the impact of these network dimensions on bonding and bridging social capital. H7 and H8 examine the roles of network size and diversity in the process of identity construction. Figure 1 depicts these pathways and the fundamental hypotheses of the study.

This integrative framework offers a sophisticated perspective to comprehend the Facebook experiences of Bangladeshi women as a domain of negotiation among structure, agency, and identity. It underscores that social media is not solely a communication instrument but a cultural arena where power, norms, and representation intersect.

## 3 Methodology

### 3.1 Research design

This research utilized an explanatory sequential mixed-methods design (Creswell and Plano Clark, 2017) to investigate the impact of Facebook involvement on the formation of social capital and identity construction among Bangladeshi women. Facebook was selected as the primary platform for analysis because of its extensive utilization across various generational and geographic demographics in Bangladesh, especially among women (Chowdhury N. J. and bin Ahsan, 2024; Chowdhury N. and bin Ahsan, 2024). In contrast to single-function applications such as WhatsApp or entertainment-centric platforms like TikTok, Facebook provides a multifaceted environment that integrates timeline posts, private messaging, groups, and audience control features. This structure enables both public self-presentation and private identity negotiation (Hoffbauer, 2012; Smock et al., 2011). These features render Facebook especially appropriate for analyzing the dual aspects of social capital and identity formation within a gendered framework. Future research should consider examining additional platforms, including WhatsApp and TikTok, which may fulfill distinct communicative and relational roles. The research was executed in two stages. The quantitative phase comprised a cross-sectional survey that evaluated Facebook usage, network attributes, social capital metrics, and identity-related factors. Subsequently, a qualitative phase involved semi-structured interviews to elucidate the statistical trends identified in the survey. This strategy adheres to an explanatory framework, wherein quantitative findings influence qualitative exploration, facilitating methodological triangulation and a more profound comprehension of the phenomena (Chan, 2018; Palinkas et al., 2015). The survey identifies generalizable trends and examines theoretical relationships using Structural Equation Modeling (SEM), while the interviews provide context, illuminating the lived experiences, strategies, and subjective interpretations of digital behaviors in a gendered, non-Western setting.

### 3.2 Sampling and participants

The study focused on adult Bangladeshi women who regularly use Facebook, intending to capture varied experiences across age, geographic location, and digital engagement. A total of 357 female participants were recruited using purposive and snowball selection methods, assuring variety and relevance. Recruitment commenced via women-centric networks, NGOs, and digital community platforms, supplemented by referrals to ensure the inclusion of participants from rural and semi-urban areas. Participants were mandated to fulfill the subsequent eligibility criteria: (1) be a minimum of 18 years old, (2) identify as female, and (3) engage with Facebook at least once weekly. This threshold may indicate moderate rather than heavy users, although it guaranteed the inclusion of participants with consistent platform exposure, while still capturing the diverse intensity of usage patterns among Bangladeshi women. The final sample encompassed a broad spectrum of Bangladeshi society regarding age, education, religion, and occupation. The age distribution adhered to four specified cohorts in the questionnaire: 22–29, 30–39, 40–49, and 50 years or older, with the majority predominantly in the 30–49 group. Facebook usage habits revealed generational differences, with younger women exhibiting more expressive and experimental activities, while older users prioritized familial connections and informational purposes.

Participants were categorized as urban or rural according to self-reported location. Urban users frequently utilized Facebook for networking, professional advancement, and community involvement, whereas rural users mostly employed the platform for maintaining familial connections and obtaining supplies. Educational attainment was significantly elevated within the sample: roughly 62% possessed Master’s degrees, 36% held Bachelor’s degrees, and around 2% had or were pursuing doctoral qualifications. This distribution illustrates current patterns in Facebook use, which are closely linked to educational access and digital literacy in Bangladesh (Mamun, 2024). This sample accurately represents the digital behaviors of educated and urban Bangladeshi women, but it may not reflect those of rural or less educated users with restricted access to technology. Our findings cannot be applied to all Bangladeshi women due to this demographic bias.

To augment qualitative depth and triangulate findings, 15 individuals were chosen from the survey pool for follow-up semi-structured interviews employing maximum variation sampling (Palinkas et al., 2015). In the qualitative phase, 15 participants were chosen using purposive sampling to guarantee variety in age, marital status, educational attainment, and frequency of Facebook usage. The number was considered adequate according to the principle of data saturation, as no new themes surfaced following the twelfth interview. The interviews were conducted both in person and online through Zoom calls, based on participant convenience. All participants were informed about the study’s objectives and procedures and gave informed consent before participation, in compliance with ethical standards. This methodology guaranteed the representation of varied perspectives according to age, geographic region, educational background, and Facebook engagement behaviors. The amalgamation of quantitative breadth and qualitative depth facilitated a comprehensive analysis of how women from various social strata formulate identity and social capital online. The attained sample size of 357 fulfilled the minimum structural criterion for Partial Least Squares Structural Equation Modeling (PLS-SEM), adhering to the established “10-to-1” indicator-to-path guideline (Hair et al., 2019). Absolute confidentiality and anonymity were upheld, especially considering the societal sensitivities related to women’s online speech in Bangladesh.

### 3.3 Quantitative measures and instruments

The quantitative phase utilized a structured, bilingual questionnaire (English and Bengali) aimed at assessing Facebook usage, social network characteristics, dimensions of social capital, and identity construction. All items were derived from validated international scales and refined via localization procedures to ensure cultural relevance for Bangladeshi women. A pilot test involving ten participants was conducted before deployment to assess clarity and contextual fit, resulting in minor linguistic and layout modifications. All constructs were assessed using 5-point Likert scales such as, with responses ranging from 1 (Strongly Disagree) to 5 (Strongly Agree), facilitating uniform psychometric evaluation across variables. A 5-point Likert scale was utilized for all items to improve clarity and respondent understanding, as this format is well-established for its cultural relevance and interpretative simplicity in survey research within South Asian contexts. Table 1. presents a summary of the key constructs assessed in this study, detailing the number of items, representative indicators, and the original sources from which the items were adapted or adopted.

**Table 1:** Measurement of constructs and items.

Construct	Sample items	Source
Facebook intensity	“Facebook is part of my everyday activity”	Adapted from Ellison et al. (2007)
Bonding social capital	“I trust my family members, neighbors, and community members”	Adapted from Williams (2006) and Williams (2019)
Bridging social capital	“Most of the organizations represent my interests.”	Adapted from Williams (2006) and Ahmad et al. (2022)
Identity construction	“I know exactly what I feel and want.”	Adapted from Cheung and Lee (2010) and Mei and Symaco (2022)
Network size	“I talk to a lot of people every day.”	Self-constructed based on Lin (2001) and Putnam (2000)
Network diversity	“My personal and social circle includes people of different races and ethnicities.”	Adapted from Putnam (2000) and Lin (2001)

The measurement of Facebook engagement utilized the Facebook Intensity Scale, as established by Ellison et al. (2007). This scale evaluates emotional connection, behavioral involvement, and the extent of Facebook’s integration into daily life. The assessment of network size and perceived connectivity utilized modified items from the Social Network Index (Cohen et al., 1997), which measured both the volume and activity of social ties. Network diversity was defined using measures that assess heterogeneity across various demographic and ideological dimensions, including age, occupation, religion, and political beliefs, in accordance with Cohen et al.’s framework. The assessment of bonding social capital utilized items modified from the Personal Social Capital Scale (Chen et al., 2009) and Williams (2006), emphasizing trust, reciprocity, and emotional closeness within strong ties. Bridging social capital was assessed through the Internet Social Capital Scales (Williams, 2006), highlighting the importance of exposure to diverse perspectives and engagement in extensive social networks and organizational activities. Identity construction was assessed using modified items from the Dimensions of Identity Development Scale (Luyckx et al., 2008), which addressed elements of self-concept clarity, self-expression, and internal harmony enabled by digital interaction.

All scale items exhibited high internal consistency, with Cronbach’s alpha values between 0.866 and 0.958, signifying excellent reliability across constructs. The application of standardized, theoretically informed instruments, along with cultural adaptation, guaranteed that the data gathered were both reliable and contextually relevant. This method facilitated a quantitative analysis of the complex relationship among Facebook usage, network composition, social capital accumulation, and identity formation within the sociocultural context of Bangladeshi women.

### 3.4 Data analysis strategy

We utilized Partial Least Squares Structural Equation Modeling (PLS-SEM) using SmartPLS 4 to evaluate the proposed linkages in our conceptual model. PLS-SEM was chosen for its several methodological advantages. Initially, it is adept at assessing intricate models comprising numerous structures and indicators. Secondly, it mandates minimal criteria for data distribution and sample size, rendering it suitable for our dataset, which displayed minor departures from normalcy. Third, PLS-SEM is proficient for models with both exploratory and confirmatory aims, since it emphasizes the maximization of explained variance in the dependent variables (Benitez et al., 2020; Hair et al., 2019).

### 3.5 Data analysis procedures

The data analysis was performed in two consecutive phases, consistent with the mixed-methods research design. In the quantitative phase, survey data (N = 357) were examined utilizing Partial Least Squares Structural Equation Modelling (PLS-SEM) with SmartPLS 4.0. PLS-SEM was selected for its efficacy in modelling intricate interactions and its ability to handle non-normal data distributions in moderate sample sizes (Benitez et al., 2020; Hair et al., 2019). Prior to analysis, the dataset was examined for absent values. No substantial missing data were detected; hence, all cases were preserved, and no imputation methods were required. The analysis comprised a two-step process: (1) review of the measuring model for reliability and validity, and (2) assessment of the structural model to examine the proposed links.

The evaluation of the measurement model encompassed internal consistency reliability (Cronbach’s alpha, Composite Reliability), convergent validity (Average Variance Extracted), and discriminant validity assessed using the Heterotrait–Monotrait (HTMT) ratio. Following the verification of the psychometric integrity of the components, the structural model was evaluated to assess path coefficients (*β*), their statistical significance (via bootstrapping with 5,000 subsamples), and the variance explained (R^2^) for the endogenous constructs. These metrics offered insight into the explanatory capacity and theoretical significance of the proposed model paths.

The qualitative component involved transcribing semi-structured interviews with 15 participants verbatim and conducting thematic analysis in accordance with Braun and Clarke’s (2006) six-phase approach. Inductive and deductive coding strategies were utilized to identify patterns concerning digital self-presentation, identity negotiation, trust-building, and social resource mobilization. NVivo software was employed to systematically organize and code the textual data. An impartial co-coder evaluated a portion of the transcripts to validate the thematic coding method. Inter-coder agreement was evaluated through collaborative analysis and iterative development of themes, ensuring interpretative consistency and reducing subjective bias. The qualitative findings enriched and contextualized the quantitative results, providing a nuanced interpretation of how Bangladeshi women navigate Facebook as a platform for identity construction and social capital cultivation within a gendered sociocultural framework.

## 4 Result of the study

### 4.1 Descriptive statistics of the sample

The sample included 357 participants, predominantly aged 30 to 49. Of the participants, 35.9% were in the age group of 30–39 years, while 39.2% were in the age group of 40–49 years. Participants aged 22–29 constituted 19.9% of the sample, whereas those aged 50 and above accounted for only 5.0%. Of the participants, 53.2% reported residing in rural areas, whereas 46.8% were from urban regions. The sample exhibited a high level of educational attainment, with 61.9% holding a master’s degree, 36.1% having completed a bachelor’s degree, and 2.0% possessing a doctorate. The data indicate a largely educated population, pertinent for analyzing outcomes associated with digital literacy, social capital, and identity formation (see Table 2).

**Table 2:** Demographic characteristics of participants.

Variable	Category	Count	Column N %
Age	22–29	71	19.9%
	30–39	128	35.9%
	40–49	140	39.2%
	50 or above	18	5.0%
Locality	Urban	167	46.8%
	Rural	190	53.2%
Education level	Bachelor	129	36.1%
	Master	221	61.9%
	Doctorate	7	2.0%

### 4.2 Measurement model evaluation (PLS-SEM)

The analysis of the measurement model in this study sought to determine the construct validity and reliability of the latent variables: Bonding Social Capital (BSC), Bridging Social Capital (BrSC), Facebook Involvement (FI), Identity Construction (IC), Network Size (NS), and Network Diversity (ND). In the PLS-SEM methodology, we initially evaluated factor loadings, which reflect the intensity of the association between observable variables and their corresponding latent constructs.

Table 3 indicates that all factor loadings surpassed the recommended threshold of 0.70, thereby affirming robust convergent validity among the constructs. This suggests that the items observed consistently reflect their corresponding theoretical constructs. The item loadings for Bonding Social Capital (0.852–0.905) and Bridging Social Capital (0.792–0.861) substantiate the effectiveness of assessing both intimate connections and wider, diverse relationships facilitated by Facebook. Items related to Facebook Involvement (0.820–0.864) indicate the extent to which the platform is woven into participants’ social habits, whereas Identity Construction items (up to 0.881) illustrate how Facebook influences self-expression and the evolution of identity. The metrics of Network Size (0.777–0.850) and Network Diversity (0.905–0.946) provide additional evidence of the instrument’s capacity to evaluate users’ social reach and the variety of their contacts. The results strongly substantiate the construct validity and reliability of the measurement model. They establish that Facebook operates as both a connector and a catalyst for social capital and identity negotiation, particularly for women maneuvering through cultural constraints in patriarchal environments. This validated model provides a robust empirical basis for analyzing the transformative impact of digital platforms in non-Western societies.

**Table 3:** Factor loadings.

Item code	Bonding social capital	Bridging social capital	Facebook involvement	Identity construction	Network size	Network diversity
BSC1	0.856					
BSC2	0.878					
BSC3	0.905					
BSC4	0.862					
BSC5	0.853					
BrSC1		0.81				
BrSC2		0.848				
BrSC3		0.861				
BrSC4		0.856				
BrSC5		0.792				
FI1			0.82			
FI2			0.825			
FI3			0.846			
FI4			0.839			
FI5			0.864			
IC1				0.863		
IC2				0.869		
IC3				0.735		
IC4				0.866		
IC5				0.881		
ND1						0.946
ND2						0.913
ND3						0.942
ND4						0.921
ND5						0.905
NS1					0.788	
NS2					0.777	
NS3					0.85	
NS4					0.81	
NS5					0.827	

### 4.3 Convergent and discriminant validity

In order to assess the impact of Facebook on social capital and identity among Bangladeshi women, it is crucial to confirm that the measurement scales employed in the study exhibit both reliability and validity. The assessment of construct validity involved examining convergent validity and discriminant validity, which are essential elements for evaluating the sufficiency of the latent constructs: Bonding Social Capital, Bridging Social Capital, Facebook Involvement, Identity Construction, Network Size, and Network Diversity.

Table 4 indicates that all constructs exhibited robust internal consistency, as evidenced by Cronbach’s alpha values surpassing the 0.70 threshold. Bonding Social Capital recorded an alpha of 0.92, while Bridging Social Capital achieved 0.89, and Facebook Involvement reached 0.895. The composite reliability scores further validated the reliability of the scales, indicating values of 0.887 for Network Size and 0.959 for Network Diversity. The findings on convergent validity were reinforced by Average Variance Extracted (AVE) values exceeding 0.50 for all constructs, demonstrating that each construct accounts for more than half of the variance in its indicators. Network Diversity and Bonding Social Capital demonstrated AVEs of 0.857 and 0.759, respectively, indicating strong evidence of item convergence within the constructs.

The assessment of discriminant validity was conducted through the Heterotrait–Monotrait ratio (HTMT) for discriminant validity, which involves comparing the square root of the AVE for each construct against its correlations with other constructs. The results indicated that each construct exhibited a higher degree of variance with its respective indicators compared to any other construct within the model. For example, Bonding and Bridging Social Capital, while both representing dimensions of social capital, were found to be statistically distinct, as were Network Size and Network Diversity. In a similar vein, Facebook Involvement and Identity Construction were empirically distinguished, highlighting their conceptual distinctiveness. The measurement model exhibited robust reliability and validity, affirming that the constructs effectively capture the fundamental dimensions of Facebook use, social capital, and identity construction among Bangladeshi women. The psychometric properties establish a robust basis for understanding the structural relationships outlined in the following analysis.

**Table 4:** Convergent validity and reliability.

Construct	Cronbach’s alpha	Composite reliability (rho_a)	Composite reliability (rho_c)	Average variance extracted (AVE)
Bonding social capital	0.92	0.92	0.94	0.759
Bridging social capital	0.89	0.894	0.919	0.695
Facebook involvement	0.895	0.897	0.922	0.704
Identity construction	0.898	0.901	0.925	0.713
Network size	0.872	0.887	0.905	0.657
Network diversity	0.958	0.959	0.968	0.857

### 4.4 Discriminant validity: HTMT ratio

Discriminant validity was evaluated utilizing the Heterotrait- Monotrait (HTMT) ratio (Henseler et al., 2015), which are recognized methods in PLS-SEM for ascertaining construct distinctiveness. HTMT ratios were analyzed to enhance the validation of the distinctiveness across components. All HTMT values Table 5 were below the stringent criterion of 0.85, offering robust evidence of discriminant validity. Collectively, these findings confirm that the latent variables inside the model are both theoretically and empirically distinct.

**Table 5:** Heterotrait–Monotrait Ratio (HTMT) for discriminant validity.

Latent construct	Bonding social capital	Bridging social capital	Facebook involvement	Identity construction	Network size	Network diversity
Bridging social capital	0.724					
Facebook involvement	0.743	0.634				
Identity construction	0.723	0.647	0.644			
Network size	0.762	0.546	0.603	0.654		
Network diversity	0.717	0.682	0.77	0.59	0.585	

The HTMT ratios presented in Table 5 remain below the conservative threshold of 0.85 (Henseler et al., 2015), reinforcing the distinctiveness of the measured constructs. The findings suggest that Bonding Social Capital, Bridging Social Capital, Facebook Involvement, Network Size, Network Diversity, and Identity Construction are distinct variables both empirically and conceptually. The presence of strong convergent and discriminant validity reinforces the reliability of the measurement model, establishing a solid basis for the further assessment of structural paths and hypothesis testing.

### 4.5 Structural model evaluation

Upon establishing the reliability and validity of the measurement model, the structural model was evaluated to examine the proposed relationships among the constructs. The evaluation comprised an examination of path coefficients, the coefficient of determination (R^2^), Bootstrapping with 5,000 samples was employed to determine the significance of the path relationships. The explanatory power and model fit of the structural model were evaluated by examining R-squared (R^2^) and Standardized Root Mean Square Residual (SRMR) values. The R^2^ value represents the extent to which the predictors account for the variance in each endogenous construct, whereas the SRMR offers a measure of the model’s fit. Table 6 illustrates the extent to which Facebook usage serves as a predictor for variations in social capital dimensions, identity construction, and network attributes among Bangladeshi women.

**Table 6:** R square and SRMR.

Dependent variable	R-square	R-square adjusted	SRMR
Bonding social capital	0.613	0.611	0.093
Bridging social capital	0.434	0.431	
Identity construction	0.429	0.425	
Network size	0.307	0.305	
Network diversity	0.512	0.51	

The findings indicate that Bonding Social Capital achieved the highest R^2^ (0.613), implying that Facebook plays a significant role in enhancing close social connections. The analysis of Network Diversity revealed a significant R^2^ value of 0.512, suggesting that Facebook significantly contributes to broadening users’ access to a variety of social connections. The relationship between Social Capital and Identity Construction demonstrated a moderate level of explanatory power (R^2^ ≈ 0.43), underscoring the role of Facebook in enhancing wider social connections and personal expression. The R^2^ for Network Size was lower at 0.307, yet it remains significant in illustrating the platform’s influence on social reach. The SRMR value of 0.093 is within acceptable limits, indicating a favorable model fit. Henseler et al. (2015) assert that an SRMR value under 0.10 is deemed acceptable for PLS-SEM, especially in exploratory models. Consequently, the resulting SRMR = 0.093 is comfortably inside the suggested threshold, affirming the model’s goodness-of-fit. The collective findings bolster the model’s credibility in elucidating the role of Facebook engagement in enhancing women’s social connectivity and identity formation within a digitally mediated environment.

In order to assess the proposed connections among Facebook usage, social capital, and identity construction, we utilized Partial Least Squares Structural Equation Modeling (PLS-SEM) with bootstrapping, conducting 5,000 resamples. In this context, hypothesis testing facilitates the statistical validation of the proposed conceptual model by analyzing the strength, direction, and significance of each path coefficient. Table 7 illustrates the testing of eight hypotheses, each representing a direct relationship among Facebook involvement, network attributes, social capital, and identity outcomes in the context of Bangladeshi women.

**Table 7:** Hypothesis testing results.

Hypothesis	Original sample (O)	Sample mean (M)	Standard deviation (STDEV)	T statistics (|O/STDEV|)	*p* values
H1	0.715	0.717	0.034	20.774	<0.001
H2	0.554	0.555	0.042	13.064	<0.001
H3	0.411	0.411	0.045	9.123	<0.001
H4	0.511	0.514	0.056	9.196	<0.001
H5	0.314	0.314	0.059	5.314	<0.001
H6	0.314	0.314	0.046	6.826	<0.001
H7	0.221	0.223	0.047	4.697	<0.001
H8	0.427	0.429	0.054	7.847	<0.001

All proposed pathways (H1–H8) received statistical validation, with *p*-values < 0.001, affirming the model’s robustness. The most robust correlation was identified in H1 (*β* = 0.715, t = 20.774), suggesting that increased Facebook activity is positively associated with greater network diversity. While structural equation modeling facilitates the examination of directional hypotheses, it is important to interpret the results as associative rather than definitively causal. Likewise, H2 (β = 0.554, t = 13.064) demonstrates that heightened Facebook engagement is positively associated with larger network size. Network diversity (H4, β = 0.511) and network size (H6, β = 0.314) were significant predictors of bonding social capital, whereas diversity (H4) also highly predicted bridging social capital (H4, β = 0.511). These findings confirm the pivotal function of Facebook in fostering both forms of social capital. Both network diversity (H5, β = 0.314) and network size (H8, β = 0.427) were positively correlated with identity formation, indicating that extensive and many social connections on Facebook facilitate women’s online identity development. Despite H7 (network size → bridging social capital) exhibiting a comparatively lower coefficient (β = 0.221), it retained statistical significance, highlighting the intricate relationship between network number and quality in the development of digital social capital.

**Figure 2:**
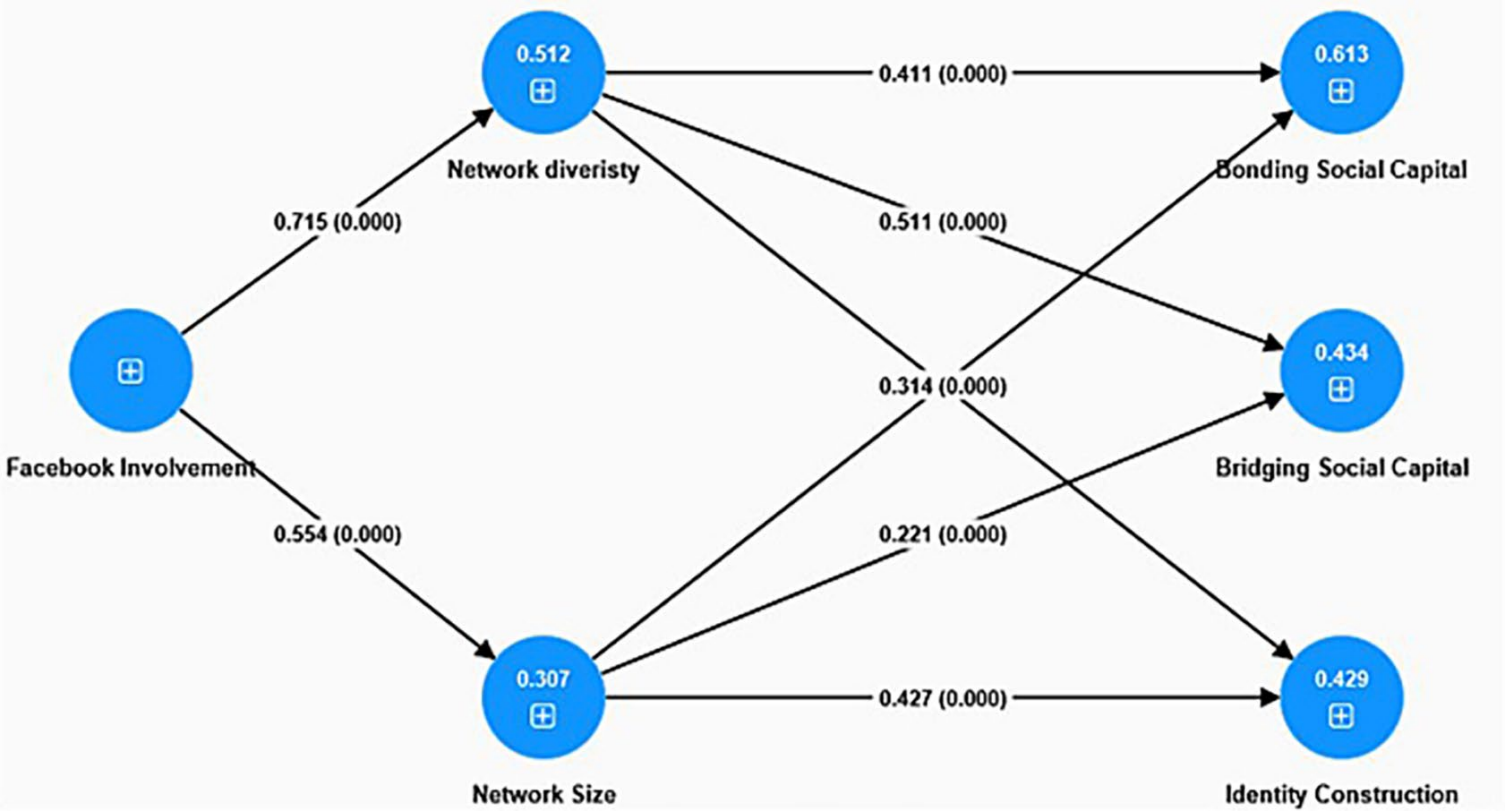
Final structural model of Facebook use, Social capital, and identity construction among Bangladeshi women.

Figure 2 conclusive structural model of Facebook utilization, social capital, and identity formation among Bangladeshi women. Arrows indicate important proposed relationships between underlying constructs. Next to each arrow, the standardized path coefficients (β) are displayed. The R2 values indicate the proportion of variance in each endogenous variable that is accounted for by the model. Every path demonstrated statistical significance at p < 0.001. The model assesses the theoretical framework that connects Facebook involvement to network expansion, social capital formation, and identity construction. The final structural model substantiates the primary assertion that participation in Facebook plays a significant role in the enhancement of social capital and identity among Bangladeshi women. The use of Facebook is a significant predictor of both network diversity (β = 0.715) and network size (β = 0.554), highlighting its influence on the expansion of women’s digital social reach. The expansion of these networks contributes to an increase in bonding (R2 = 0.613) and bridging social capital (R2 = 0.434), thereby affirming Putnam’s dual social capital framework within a non-Western, gendered context.

Both network diversity and size significantly influence identity construction (R^2^ = 0.429), underscoring Facebook’s role as a crucial platform for self-expression and identity negotiation. The results reinforce the incorporation of Social Capital Theory, Social Identity Theory, and Goffman’s dramaturgical model, while furthering feminist digital sociology by illustrating the ways in which women manage visibility and limitations in online environments. In conclusion, the model demonstrates that digital platforms function beyond mere communication tools; they serve as socio-cultural arenas where identity and agency are actively constructed.

### 4.6 Qualitative insights: negotiating identity and social capital on Facebook

Qualitative data from 15 semi-structured interviews enhanced the quantitative findings by elucidating how Bangladeshi women perceive and maneuver identity and social capital on Facebook. Participants described Facebook as a dual-faceted environment that is both empowering and constraining. A participant stated, “Facebook allows me to express myself freely in my women’s group, but it’s too risky for me to share those thoughts on my public timeline,” Another individual remarked, “I use a different name in private groups to seek help or discuss sensitive topics that my family cannot know about.” These insights illustrate how women strategically utilize platform features to preserve agency while navigating conservative cultural norms, reflecting the wider dynamics of identity formation and social capital development in digital environments. For numerous individuals, women-exclusive groups and selective visibility configurations offered a digital sanctuary for personal narrative, support-seeking, or norm-challenging, hence enhancing the significance of platform affordances in fostering bridging capital. Concurrently, robust connections with family and community evidenced by regular online interactions paralleled the quantitatively assessed bonding capital. Thematic themes like “strategic self-presentation,” “digital sisterhood,” and “negotiated authenticity” underscored how women meticulously navigated cultural conformity and personal expression. These narratives corroborate the structural model’s conclusion that Facebook activity and network diversity are major predictors of both bonding and bridging capital, which subsequently affect identity formation.

## 5 Discussion

This research aimed to examine the impact of Facebook usage on social capital and identity formation among women in Bangladesh, using Social Capital Theory, Social Identity Theory, and Goffman’s dramaturgical perspective through a mixed-methods methodology. The results demonstrate that Facebook functions as both a connected framework and a performative platform, where social capital is developed and identity is negotiated within the limitations of a patriarchal digital society.

### 5.1 Social Capital in Context: bonding and bridging dimensions

The findings clearly support the significance of Social Capital Theory (Lin, 2001; Putnam, 2000) in contemporary society, illustrating how Facebook aids in the cultivation of both bonding and bridging capital among Bangladeshi women. The emergence of bonding social capital stands out as the most significant outcome, evidenced by the highest explained variance (R^2^ = 0.613). This underscores the importance of Facebook in sustaining emotionally close, trust-based relationships, particularly in a context where women encounter mobility restrictions and limited opportunities for offline interaction. In alignment with previous research (e.g., Burke and Kraut, 2014; Ellison et al., 2007), our qualitative data support the notion that for numerous participants, Facebook serves as an essential connection to family and kinship networks. Nonetheless, the platform facilitates the development of bridging social capital though to a lesser extent mainly through the diversity of networks rather than their size (*β* = 0.511 vs. β = 0.314). Our study participants agreed: while many had enormous Facebook friend networks, just a small percentage were personally meaningful, reinforcing saturation without depth. This indicates that interaction with diverse social circles, rather than the mere quantity of contacts, facilitates women’s access to new information and perspectives, aligning with Granovetter’s (1973) theory of weak ties and modern digital interpretations (Phua et al., 2017; Tasnima et al., 2024). The importance of diverse group participation, such as women’s entrepreneurial collectives and health forums, was consistently highlighted in interviews. This indicates that digital inclusion initiatives should extend beyond mere access to also strengthening cross-cutting connections (Fatima et al., 2025; Salam, 2020).

The findings enhance Social Capital Theory by highlighting the cultural factors that influence the activation and advantages of social capital. In the context of Bangladesh, characterized by collectivism and gender stratification, bonding capital provides emotional security while simultaneously reinforcing traditional norms. Several women indicated a sense of being monitored within close family networks, which limited their ability to express themselves genuinely an occurrence that aligns with research on digital self-regulation in patriarchal contexts (Klose and Jebin, 2024). On the other hand, bridging capital presents opportunities for empowerment; however, access to it is inconsistent, influenced by disparities in digital literacy and apprehension regarding social repercussions, especially among rural or less-educated individuals (Hakimi et al., 2024). This underscores the necessity for strategies that promote digital confidence and ensure safe, diverse environments.

### 5.2 Social identity dynamics in the digital sphere

Our findings substantiate the relevance of Social Identity Theory (Tajfel and Turner, 1979) by demonstrating the ways in which online group membership, comparative processes, and the adoption of norms influence identity formation among female users. The research demonstrated that women adeptly navigated various identities in response to the perceived audience, reflecting a context-dependent self-categorization aligned with Social Identity Theory. For example, aligning oneself with feminist or entrepreneurial communities in private Facebook groups provided participants with a sense of empowerment and the internalization of new roles, frequently differing from the identities expressed on public timelines. This dual identity performance illustrates the fluid nature of digital selfhood, as discussed in recent SIT applications (Choi, 2024; Mei and Symaco, 2022). It is noteworthy that the affirmative path coefficients from network diversity and bridging capital to identity construction (R^2^ = 0.429) indicate that engagement with diverse groups serves as a significant catalyst for the transformation of identity. Participants frequently articulated a sense of being “seen” or “validated” within these groups, underscoring the notion that digital environments can promote identity transformations through collective self-assessment (Banet-Weiser, 2018; Luyckx et al., 2008). Digital interactions within groups are progressively seen as foundational to the development of civic and activist identities, especially in constrained physical contexts (Hong and Kim, 2021).

### 5.3 Goffman’s dramaturgy in a digital context

Goffman’s (1959) dramaturgical model offers a compelling framework for analyzing the deliberate self-presentation behaviors exhibited by individuals. The participants in our research meticulously crafted their public personas on Facebook, conforming to socially sanctioned ideals highlighting familial commitments, religious devotion, or scholarly achievements while relegating divergent opinions, personal insecurities, or sensitive subjects to more private spaces such as pseudonymous accounts or restricted groups. This is consistent with prior studies on digital impression management (Labben, 2022; Rui and Stefanone, 2013) and reinforces the growing body of evidence indicating that women’s online engagement involves emotional negotiation stemming from privacy concerns and relational anxiety (Hu et al., 2024). This underscores the manner in which digital environments mirror real-world narratives, necessitating ongoing audience consciousness and emotional investment from underrepresented individuals. The convergence of SIT and Goffman’s theory is notably significant: women who participated in bridging behavior frequently had to traverse the complexities of identity fragmentation, balancing various community norms while protecting themselves from possible backlash by sustaining multiple online personas. This reinforces the idea that digital identity is complex and is curated through intentional expressions across various social strata (Couldry and Mejias, 2019).

### 5.4 Convergence of theoretical frameworks

The combined application of Social Capital Theory, Social Identity Theory, and Goffman’s dramaturgy yields significant analytical benefits. The SEM findings validate that Facebook usage enhances network structures (social capital), which subsequently influence self-perception (identity), while qualitative data illustrate how these processes are moderated by performative self-regulation. The interaction among network access, group affiliation, and impression management highlights the complex character of digital empowerment, wherein identity construction is both facilitated and restricted by socio-technical systems.

### 5.5 Implications of feminist digital sociology and ICT4D

This study, from a feminist digital sociology viewpoint, elucidates how digital affordances both empower and constrain women’s engagement in the public realm (Banet-Weiser, 2018; Wajcman, 2010). Participants utilized Facebook to engage in entrepreneurship, political awareness, education, and support networks demonstrations of digital agency (Fatima et al., 2025; Mamun, 2024). Nonetheless, they encountered continual surveillance, gendered inspection, and the necessity to exhibit respectability, illustrating the adaptability of patriarchal standards within digital platforms.

The findings indicate that for the ICT4D community, digital inclusion must extend beyond mere infrastructure or access. Digital literacy training, secure group design, and platform accountability are essential for facilitating meaningful participation (Chew et al., 2015). The utilization of Facebook groups for peer mentorship, informal education, or health assistance indicates scalable opportunities for developmental interventions. The research highlights the capacity of Facebook groups to function as platforms for peer mentorship, informal education, and emotional support, which are particularly crucial in digitally constrained, patriarchal societies (Banet-Weiser, 2018; Chew et al., 2015; Fatima et al., 2025). However, genuine impact necessitates confronting structural disparities and societal norms that persistently influence women’s digital habits. This study conclusively demonstrates that Facebook functions as both a catalyst and a repository for the accumulation of social capital and the creation of identity among Bangladeshi women. It corroborates established theories while providing innovative perspectives on the interplay between gendered socio-cultural contexts and platform design in influencing digital involvement. The results underscore the significance of network diversity, group affiliation, and impression management in comprehending the role of digital technology in facilitating empowerment and imposing constraints. As social media increasingly influences civic and social life, detailed, context-specific research such as this is vital to guarantee that connectedness fosters inclusiveness rather than simply surveillance or self-censorship.

### 5.6 Limitation of the study

This research has several limitations. First, although there was balanced representation in both urban and rural areas, the sample was primarily composed of highly educated women, which may have limited the findings’ applicability to less literate or socioeconomically marginalized groups. Second, although the structural model used PLS-SEM to examine direct associations, it did not investigate mediation effects, which limited understanding of underlying mechanisms. Future research should include mediators to find more complex pathways. Third, the connections found should be regarded as correlational rather than causal because the cross-sectional design makes it impossible to draw conclusions about causality. Lastly, despite being rich in contextual insights, the qualitative sample (n = 15) might not accurately represent the variety of digital experiences that Bangladeshi women have, necessitating larger samples in subsequent qualitative studies.

## 6 Conclusion

This study provides a theoretical examination of Facebook’s role in fostering social capital and negotiating identity among Bangladeshi women. Utilizing Social Capital Theory, Social Identity Theory, and Goffman’s dramaturgical framework, alongside a comprehensive mixed-methods approach, we illustrate that Facebook transcends its role as a connection tool; it serves as a culturally ingrained social environment where gendered agency, performance, and resistance manifest. The study quantitatively establishes that Facebook usage markedly improves both bonding and bridging social capital, with network diversity identified as a more potent predictor of identity formation than network size alone. Women’s online interactions expanded their access to ideas, networks, and support systems that were otherwise challenging to obtain in a patriarchal culture. Nonetheless, these advantages are not equitably allocated. Structural and cultural impediments such as deficiencies in digital literacy, surveillance, and ingrained norms persist in influencing the extent to which women can utilize the platform freely. The contextual nuances are essential: whereas digital technologies have the capacity to redistribute power and broaden identity options, they may also perpetuate existing social hierarchies through subtle control mechanisms.

Our findings qualitatively demonstrate how women strategically navigate their online presence and construct digital identities. Facebook serves as a platform where women enact idealized personas to satisfy audience expectations, while concurrently establishing private places for more genuine or rebellious expression. This dual navigation illustrates the empowering possibilities and regulatory constraints of social media under conservative cultural contexts.

Theoretically, the research enhances current frameworks by demonstrating how social capital facilitates identity transformation via digitally mediated group affiliation and performance. It enhances feminist digital sociology by demonstrating that online platforms serve as both venues of empowerment and loci of gendered limitation, especially in the Global South. Furthermore, the amalgamation of structural, cognitive, and performative aspects of digital connection provides a comprehensive framework for comprehending women’s social media behaviors in non-Western settings.

The study indicates that policy measures designed to improve women’s digital participation should extend beyond merely providing infrastructure. This study enhances the understanding of Facebook as a gendered platform for the construction of social capital and identity negotiation among Bangladeshi women. Utilizing Social Capital Theory, Social Identity Theory, and Goffman’s dramaturgical perspective, the findings demonstrate that digital environments present both opportunities and limitations influenced by cultural norms and platform characteristics. Facebook enables novel modes of connection and self-expression; however, it simultaneously reflects existing offline inequalities and societal pressures. This research enhances feminist digital sociology and provides a culturally informed perspective on mediated identity formation in the Global South.

## Data Availability

The raw data supporting the conclusions of this article will be made available by the authors, without undue reservation.

## References

[ref1] Ahmad Z. Soroya S. H. Mahmood K. (2022). Bridging social capital through the use of social networking sites: a systematic literature review. J. Hum. Behav. Soc. Environ. 33, 473–489. doi: 10.1080/10911359.2022.2064025

[ref2] Banet-Weiser S. (2018). Empowered: Popular feminism and popular misogyny. Durham, NC: Duke University Press.

[ref3] Benitez J. Henseler J. Castillo A. Schuberth F. (2020). How to perform and report an impactful analysis using partial least squares: guidelines for confirmatory and explanatory IS research. Inf. Manag. 57:103168. doi: 10.1016/j.im.2019.05.003

[ref4] Bharathi B. (2023). Crafting digital self-navigating online identity and self-presentation. New Delhi, India: Blue Rose Publishers.

[ref9001] Braun V. Clarke V. (2006). Using thematic analysis in psychology. Qual. Res. Psychol. 3, 77–101. doi: 10.1191/1478088706qp063oa

[ref5] Burke M. Kraut R. E. (2014). Growing closer on Facebook: Changes in tie strength through social network site use. Proceedings of the SIGCHI Conference on Human Factors in Computing Systems, 4187–4196.

[ref6] Castillo de Mesa J. Gómez-Jacinto L. López Peláez A. Erro-Garcés A. (2020). Social networking sites and youth transition: the use of Facebook and personal well-being of social work young graduates. Front. Psychol. 11:230. doi: 10.3389/fpsyg.2020.00230, PMID: 32132959 PMC7040231

[ref7] Chan M. (2018). Reluctance to talk about politics in face-to-face and Facebook settings: examining the impact of fear of isolation, willingness to self-censor, and peer network characteristics. Mass Commun. Soc. 21, 1–23. doi: 10.1080/15205436.2017.1358819

[ref8] Chen X. Stanton B. Gong J. Fang X. Li X. (2009). Personal social capital scale: an instrument for health and behavioral research. Health Educ. Res. 24, 306–317. doi: 10.1093/her/cyn020, PMID: 18469318

[ref9] Cheung C. M. K. Lee M. K. O. (2010). A theoretical model of intentional social action in online social networks. Decis. Support. Syst. 49, 24–30. doi: 10.1016/j.dss.2009.12.006

[ref10] Chew H. E. Ilavarasan V. P. Levy M. R. (2015). *Mattering matters:* agency, empowerment, and mobile phone use by female microentrepreneurs. Inf. Technol. Dev. 21, 523–542. doi: 10.1080/02681102.2013.839437

[ref11] Choi D.-H. (2024). Impact of social media use on the life satisfaction of adolescents in South Korea through social support and social capital. SAGE Open 14:10. doi: 10.1177/21582440241245010

[ref12] Chowdhury J. N. bin Ahsan W. (2024). Online privacy awareness in Bangladesh: challenges and solutions. Userhub. doi: 10.58947/NYDL-LBVP

[ref13] Chowdhury N. bin Ahsan M. T. (2024). Impact of social media on body image and mental health among youth in Bangladesh: a mixed-methods study. Userhub J. 1, 1–15. doi: 10.58947/journal.fzra78

[ref14] Cohen S. Doyle W. J. Skoner D. P. Rabin B. S. Gwaltney J. M. Jr. (1997). Social ties and susceptibility to the common cold. JAMA J. Am. Med. Assoc. 277, 1940–1944. doi: 10.1001/jama.1997.03540480040036 9200634

[ref15] Couldry N. Mejias U. A. (2019). The costs of connection: How data is colonizing human life and appropriating it for capitalism. Stanford, CA: Stanford University Press.

[ref16] Creswell J. W. Plano Clark V. L. (2017). Designing and conducting mixed methods research. 3rd Edn. Thousand Oaks, CA: SAGE Publications.

[ref17] Ellison N. B. Steinfield C. Lampe C. (2007). The benefits of Facebook “friends:” social capital and college students’ use of online social network sites. J. Comput.-Mediat. Commun. 12, 1143–1168. doi: 10.1111/j.1083-6101.2007.00367.x

[ref18] Ellison N. B. Steinfield C. Lampe C. (2011). Connection strategies: social capital implications of Facebook-enabled communication practices. New Media Soc. 13, 873–892. doi: 10.1177/1461444810385389

[ref19] Fatima U. Ahmad N. Menegaki A. N. Wang H. (2025). How do information and communication technologies (ICTs) empower women? Women's Stud. Int. Forum 103:103057. doi: 10.1016/j.wsif.2025.103057

[ref20] Goffman E. (1959). The presentation of self in everyday life. New York, NY: Anchor Books.

[ref21] Granovetter M. S. (1973). The strength of weak ties. Am. J. Sociol. 78, 1360–1380. doi: 10.1086/225469

[ref22] Gudka M. Gardiner K. L. K. Lomas T. (2021). Towards a framework for flourishing through social media: a systematic review of 118 research studies. J. Posit. Psychol. 18, 1–20. doi: 10.1080/17439760.2021.1991447

[ref23] Hair J. F. Risher J. J. Sarstedt M. Ringle C. M. (2019). When to use and how to report the results of PLS-SEM. Eur. Bus. Rev. 31, 2–24. doi: 10.1108/EBR-11-2018-0203

[ref24] Hakimi M. Fazil A. W. Ahmady E. Qarizada A. Quraishi T. (2024). Cyber opportunities: fostering women’s empowerment through e-commerce in Afghanistan. Room Civil Soc. Dev. 3, 1–12. doi: 10.59110/rcsd.275

[ref25] Haythornthwaite C. (2002). Strong, weak, and latent ties and the impact of new media. Inf. Soc. 18, 385–401. doi: 10.1080/01972240290108195

[ref26] Henseler J. Ringle C. M. Sarstedt M. (2015). A new criterion for assessing discriminant validity in variance-based structural equation modeling. J. Acad. Mark. Sci. 43, 115–135. doi: 10.1007/s11747-014-0403-8

[ref27] Hoffbauer A. (2012). Book review: Zizi Papacharissi, a private sphere: democracy in a digital age. Media Cult. Soc. 34, 252–256. doi: 10.1177/0163443711431200a

[ref28] Hong H. Kim Y. (2021). What makes people engage in civic activism on social media? Online Inf. Rev. 45, 562–576. doi: 10.1108/OIR-03-2020-0105

[ref29] Hou L. (2020). Rewriting “the personal is political”: young women's digital activism and new feminist politics in China. Inter-Asia Cult. Stud. 21, 337–355. doi: 10.1080/14649373.2020.1796352

[ref30] Hu H. Shi L. Xie X. (2024). The benefit-cost balance of Chinese adults’ social interaction behaviors on social media: social capital, privacy concerns and attachment anxiety. SAGE Open 14:26. doi: 10.1177/21582440241300826

[ref31] Klose H. Jebin L. (2024). ‘I pretend to be an ideal woman just to keep their mouths shut’: Bangladeshi women’s contestation of abuse through social media platforms. Inf. Technol. Dev. 30, 246–263. doi: 10.1080/02681102.2023.2279326

[ref32] Labben A. (2022). “You’re a disgusting presenter”: collective face threat and identity positioning on Facebook. Discourse Context Media 48:100619. doi: 10.1016/j.dcm.2022.100619

[ref33] Lin N. (2001). Social capital: A theory of social structure and action. Cambridge, UK: Cambridge University Press.

[ref34] Luyckx K. Schwartz S. J. Berzonsky M. D. Soenens B. Vansteenkiste M. Smits I.. (2008). Capturing ruminative exploration: extending the four-dimensional model of identity formation in late adolescence. J. Res. Pers. 42, 58–82. doi: 10.1016/j.jrp.2007.04.004

[ref35] Mamun S. (2024). Unveiling the influence of social networks on technology adoption and entrepreneurial triumph in Bangladesh. Res. Sq. doi: 10.21203/rs.3.rs-4295549/v1

[ref36] Mei W. Symaco L. P. (2022). Students’ entrepreneurial identity construction: role and social identity influences. SAGE Open 12:961. doi: 10.1177/21582440221089961

[ref37] Page-Tan C. (2021). Bonding, bridging, and linking social capital and social media use: how hyperlocal social media platforms serve as a conduit to access and activate bridging and linking ties in a time of crisis. Nat. Hazards 105, 2219–2240. doi: 10.1007/s11069-020-04397-8

[ref38] Palinkas L. A. Horwitz S. M. Green C. A. Wisdom J. P. Duan N. Hoagwood K. (2015). Purposeful sampling for qualitative data collection and analysis in mixed method implementation research. Adm. Policy Ment. Health Ment. Health Serv. Res. 42, 533–544. doi: 10.1007/s10488-013-0528-y, PMID: 24193818 PMC4012002

[ref39] Papacharissi Z. (2015). Affective publics: Sentiment, technology, and politics. New York, NY: Oxford University Press.

[ref40] Phua J. Jin S. V. Kim J. (2017). Uses and gratifications of social networking sites for bridging and bonding social capital: a comparison of Facebook, twitter, Instagram, and snapchat. Comput. Human Behav. 72, 115–122. doi: 10.1016/j.chb.2017.02.041

[ref41] Putnam R. D. (2000). Bowling alone: The collapse and revival of American community. New York, NY: Simon & Schuster.

[ref42] Rahman R. (2023). Women empowerment in Bangladesh NGOs. Natl. Univ. J. Hum. Soc. Scie. Bus. Stud. 8, 111–126. doi: 10.3329/naujhssbs.v8i1.68084

[ref43] Raza S. A. Qazi W. Umer A. (2016). Facebook is a source of social capital building among university students: evidence from a developing country. Asian J. Commun. 26, 311–326. doi: 10.1177/0735633116667357

[ref44] Rowland J. Estevens J. (2024). “What is your digital identity?”: unpacking users’ understandings of an evolving concept in datafied societies. Media Cult. Soc. 47, 336–353. doi: 10.1177/01634437241282240

[ref45] Rui J. Stefanone M. A. (2013). Strategic self-presentation online: a cross-cultural study. Comput. Human Behav. 29, 110–118. doi: 10.1016/j.chb.2012.07.022

[ref46] Salam R. (2020). Compliance and resistance: an investigation into the construction of gender identities by Pakistani women on Facebook. Asian J. Women's Stud. 26, 503–527. doi: 10.1080/12259276.2020.1854414

[ref47] Smidi A. Shahin S. (2017). Social media and social mobilisation in the Middle East. India Q. 73, 196–209. Available at: https://www.jstor.org/stable/48505308

[ref48] Smock A. D. Ellison N. B. Lampe C. Wohn D. Y. (2011). Facebook as a toolkit: a uses and gratification approach to unbundling feature use. Comput. Human Behav. 27, 2322–2329. doi: 10.1016/j.chb.2011.07.011

[ref49] Sultana A. M. (2010). Patriarchy and women's gender ideology: a socio-cultural perspective. J. Soc. Sci. 6, 123–126. doi: 10.3844/jssp.2010.123.126

[ref50] Tajfel H. Turner J. C. (1979). “An integrative theory of intergroup conflict” in The social psychology of intergroup relations. eds. Austin W. G. Worchel S. (Monterey, CA: Brooks/Cole), 33–47.

[ref51] Tasnima T. Abu Bakar S. H. Padzil R. Md Syed M. A. (2024). It's a men's world: influence of media literacy for women's empowerment through social media engagement in Bangladesh. Inf. Dev. doi: 10.1177/02666669241303723

[ref52] Wajcman J. (2010). Feminist theories of technology. Camb. J. Econ. 34, 143–152. doi: 10.1093/cje/ben057

[ref53] Wang Y. Balnaves M. Sandner J. (2020). Shameful secrets and self-presentation: negotiating privacy practices among youth and rural women in China. SAGE Open 10:396. doi: 10.1177/2158244020903396

[ref54] Williams D. (2006). On and off the’net: scales for social capital in an online era. J. Comput.-Mediat. Commun. 11, 593–628. doi: 10.1111/j.1083-6101.2006.00029.x

[ref9002] Williams J. R. (2019). The use of online social networking sites to nurture and cultivate bonding social capital: A systematic review of the literature from 1997 to 2018. New Media Soc. 21, 2710–2729. doi: 10.1177/1461444819858749

[ref55] Yin H. Zhang Q. (2024). Digital feminism: the role of social media in shaping feminist movements in Asian cultural contexts. Adv. Hum. Res. 10, 38–42. doi: 10.54254/2753-7080/2024.18291

