# Supplementary material for: Constructing identity and social capital on Facebook: a feminist digital sociology of Bangladeshi women
Source: Front Psychol. 2025 Aug 11;16:1634395. doi: 10.3389/fpsyg.2025.1634395 (PMC12376432; doi:10.3389/fpsyg.2025.1634395)
Supplement: Supplementary file 1 [file Supplementary_file_1.pdf]

APPENDIX A

A survey tool for data collection

Questionnaire

Constructing Identity and Social Capital on Facebook: A Feminist Digital Sociology of Bangladeshi Women

Demographic Information

Gender: 1. Male 2. Female

Age: 1. 22 – 29 2. 30 – 39 3. 40 – 49 4. 50 or above

Locality: 1. Urban 2. Rural

Group: ______________________________

1. Strongly Disagree **2.** Disagree **3.** Neutral

4. Agree **5.** Strongly Agree

1. Facebook involvement

1	Facebook has become part of my daily routine.	1	2	3	4	5
2	I feel I am part of the Facebook community.	1	2	3	4	5
3	Facebook is part of my everyday activity.	1	2	3	4	5
4	I am proud to tell people I am on Facebook.	1	2	3	4	5
5	I feel out of touch when I haven't logged in to Facebook for a long time.	1	2	3	4	5

2. Network size

1	I talk to a lot of people every day.	1	2	3	4	5
2	I have a big family.	1	2	3	4	5
3	I have a large friends' circle.	1	2	3	4	5
4	I visit or talk with new people once every week.	1	2	3	4	5
5	I am involved with community members online and offline.	1	2	3	4	5

3. Network diversity

1	My personal and social circle is big.	1	2	3	4	5
2	My personal and social circle includes people of different ages.	1	2	3	4	5
3	My personal and social circle includes people of different races and ethnicities.	1	2	3	4	5
4	I have philosophy and religious-related personnel in my social circle.	1	2	3	4	5
5	I have a range of political views.	1	2	3	4	5

4. Social bonding capital

1	I have good relations with family members, neighbors, and community members.	1	2	3	4	5
2	I keep routine contact with family members, neighbors, and community members.	1	2	3	4	5
3	I trust my family members, neighbors, and community members.	1	2	3	4	5
4	My family members, neighbors, and community members will help me with my request.	1	2	3	4	5

5	My family members, neighbors, and community members have powerful resources.	1	2	3	4	5

5. Social bridging capital

1	I think organizations in my country are helpful.	1	2	3	4	5
2	I frequently join activities of different organizations.	1	2	3	4	5
3	Most of the organizations represent my interests.	1	2	3	4	5
4	Most of the organizations will help me with my request.	1	2	3	4	5
5	Most of the organizations have abundant socio-political resources.	1	2	3	4	5

6. Identity construction

1	I like myself regardless of my shortcomings.	1	2	3	4	5
2	I know exactly what I feel and want.	1	2	3	4	5
3	I have a feeling of inner harmony and peace.	1	2	3	4	5
4	I easily share my feelings with others.	1	2	3	4	5
5	I think I am becoming a better human.	1	2	3	4	5
